# Label-free multiphoton microscopy reveals relevant tissue changes induced by alginate hydrogel implantation in rat spinal cord injury

**DOI:** 10.1038/s41598-018-29140-z

**Published:** 2018-07-18

**Authors:** Roberta Galli, Kerim H. Sitoci-Ficici, Ortrud Uckermann, Robert Later, Magda Marečková, Maria Koch, Elke Leipnitz, Gabriele Schackert, Edmund Koch, Michael Gelinsky, Gerald Steiner, Matthias Kirsch

**Affiliations:** 1 Clinical Sensoring and Monitoring - Anesthesiology and Intensive Care Medicine, Faculty of Medicine, Technische Universität Dresden, Fetscherstr. 74, 01307 Dresden, Germany; 2 Molecular Neuroimaging Laboratory, Klinik und Poliklinik für Neurochirurgie, Universitätsklinikum Carl Gustav Carus, Faculty of Medicine, Technische Universität Dresden, Fetscherstr. 74, 01307 Dresden, Germany; 3 Centre for Translational Bone, Joint and Soft Tissue Research, Faculty of Medicine, Technische Universität Dresden, Fetscherstr. 74, 01307 Dresden, Germany; 4 CRTD/DFG-Center for Regenerative Therapies Dresden - Cluster of Excellence, Fetscherstr. 105, 01307 Dresden, Germany

## Abstract

The development of therapies promoting recovery after spinal cord injury is a challenge. Alginate hydrogels offer the possibility to develop biocompatible implants with mechanical properties tailored to the nervous tissue, which could provide a permissive environment for tissue repair. Here, the effects of non-functionalized soft calcium alginate hydrogel were investigated in a rat model of thoracic spinal cord hemisection and compared to lesioned untreated controls. Open field locomotion tests were employed to evaluate functional recovery. Tissue analysis was performed with label-free multiphoton microscopy using a multimodal approach that combines coherent anti-Stokes Raman scattering to visualize axonal structures, two-photon fluorescence to visualize inflammation, second harmonic generation to visualize collagenous scarring. Treated animals recovered hindlimb function significantly better than controls. Multiphoton microscopy revealed that the implant influenced the injury-induced tissue response, leading to decreased inflammation, reduced scarring with different morphology and increased presence of axons. Demyelination of contralateral white matter near the lesion was prevented. Reduced chronic inflammation and increased amount of axons in the lesion correlated with improved hindlimb functions, being thus relevant for locomotion recovery. In conclusion, non-functionalized hydrogel improved functional outcome after spinal cord injury in rats. Furthermore, label-free multiphoton microscopy qualified as suitable technique for regeneration studies.

## Introduction

Every year, between 250,000 and 500,000 people around the world suffer a spinal cord injury (SCI)^1^. SCI impacts the physical, psychological and social status of the patients and puts a substantial financial burden on them and national health care systems^2–4^. SCI results in death of neurons and destruction of white matter tracts resulting in major dysfunction of motor, sensory and autonomic systems. The primary injury to the spinal cord causes an immediate blunt or mechanical disruption of tissue structures, giving way to the secondary injury and chronic degeneration of nervous tissue and axons^5^. The subsequent events after the primary injury are a concert of complex processes, which end in tissue necrosis and degeneration leading to cavity formation, with fibrotic and glial scarring hindering axonal elongation^5^. In this context, neuroprotective and regenerative strategies aiming to minimize propagation of secondary injury and axonal degeneration are crucial. Therapeutic approaches utilizing stem cells, modulation of extracellular matrix, neurotrophic factors, as well as integrative and combinatory approaches have been studied^6–9^. Ideally, after reduction of secondary injury and creation of a regeneration-friendly environment with tissue-like mechanical properties, the axons should regrow, innervate the original target and lead to functional recovery.

Because of their hydrophilic character and biocompatibility, hydrogels became attractive for tissue engineering in a variety of tissues and organs^10^, including the nervous system^11,12^. Calcium-crosslinked alginate utilizes the electrostatic interaction of the divalent cation with the anionic polycarboxylates to form an ionically crosslinked network, which can also host drugs, growth factors, or cell grafts to sustain central nervous tissue regeneration^13,14^. The implantation of porous alginate grafts into the lesion gap in transected spinal cord of young rats has been shown to stimulate the regrowth of myelinated and unmyelinated fibers, and the formation of electrophysiological active connections^15–17^. However, contrasting results about effects of calcium alginate hydrogels on outgrowth of neurites *in vitro* were reported in the past^18,19^, while alginate implants failed to sustain axonal growth *in vivo* in absence of functionalization^20^.

The stiffness of alginate hydrogels can be tailored. This is a key factor that determines implant effects. Soft low cross-linked alginate hydrogels, with compressive modulus in the range 0.1–1.0 kPa, are ideal for application to the nervous tissue^21^. We demonstrated that soft alginate hydrogels prepared with sub-stoichiometric concentration of Ca^2+^ cations support neurite growth *in vitro* and protect neuronal cells against oxidative stress without the need of functionalization^18^. Implantation of such alginate hydrogels in small spinal cord lesions in rats improved functional recovery^22^. Moreover, they maintain their mass and volume after immersion in saline solution^23^. Indeed, a soft calcium alginate hydrogel preparation led to implants that remained stable in a rat model of SCI up to six months after injury^24,25^, with positive effects on fibrotic scarring and contralateral demyelination^25^. Other studies confirmed that ultrasoft alginate hydrogels strongly promote neuritogenesis *in vitro* and showed that they sustain the formation of functional neuronal networks^26^.

Detailed monitoring of structural and histological changes after SCI is necessary to assess the neuroprotective and regenerative effects of therapies. Optical label-free imaging techniques constitute a powerful monitoring tool besides immunohistochemistry. For instance, multiphoton microscopy offers the possibility of fast imaging of unstained tissue based on its morphochemistry. Coherent anti-Stokes Raman scattering (CARS) microscopy allows label-free imaging of lipids by selectively addressing the distribution of methylene groups in the tissue^27^, and is thus able to visualize myelin sheaths structures^28–30^. Furthermore, CARS visualizes extra- and intracellular lipid droplets^31^, for example in activated macrophages that have transformed into foam cells^32^. Two-photon excited fluorescence (TPEF) enables to identify activated inflammatory cells (microglia and macrophages) in the central nervous system^33^. Second harmonic generation (SHG) visualizes collagen type I^34^. The combination of these three multiphoton imaging techniques enables a comprehensive study of SCI^35^. Moreover, imaging with multiphoton microscopy does not require incubation and washing steps, or other processing of tissue sections. Therefore, the technique is well suited for observation of small details and delicate tissue structures that might be lost during conventional staining procedures.

We report about the effects of non-functionalized soft calcium alginate hydrogel implants in a rat model of spinal cord hemisection. The same hydrogel formulation was already proven to sustain neurite growth *in vitro*^18^ and to be stable in saline solution^23^ as well as *in vivo* up to six months after implantation^24,25^. Here, we investigated the functional outcome and used label-free multiphoton microscopy complemented by histologic techniques for detailed tissue characterization in the intermediate and chronic phase of injury. Moreover, we compared locomotor functions with tissue micro-morphology as shown by label-free multiphoton microscopy, with the aim to identify prognostic parameters and qualify this technique as diagnostic tool.

## Results

### Functional outcome

**Figure 1:**
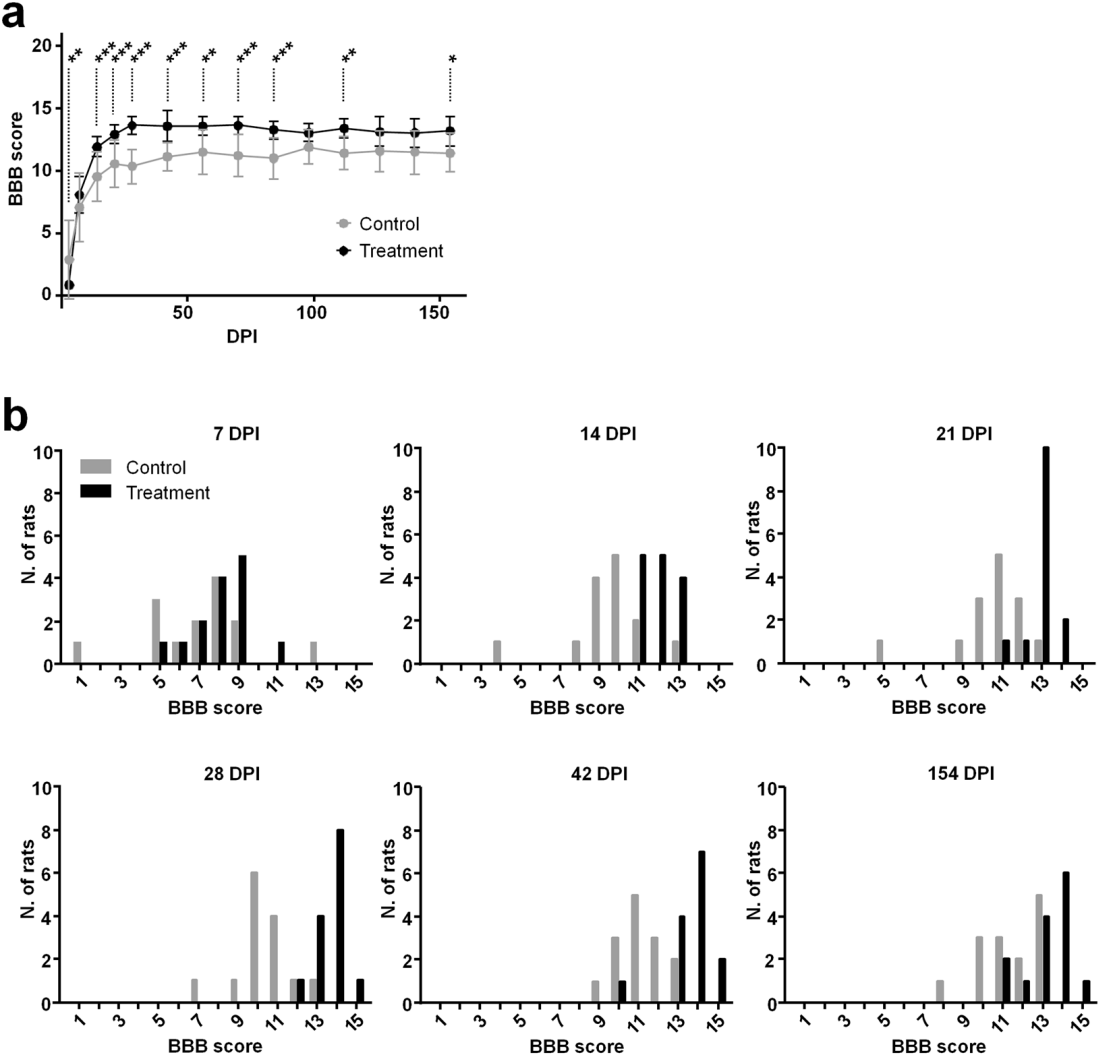
Open field behavioral tests. (**a**) Basso, Beattie and Bresnahan (BBB) scores of ipsilateral hindlimb (mean ± SD, n = 14 in each group). Bonferroni post hoc t-test, *P < 0.05, **P < 0.01, ***P < 0.001. (**b**) Frequency histograms illustrating the distribution of BBB scores at 7, 14, 21, 28, 42 and 154 DPI.

The hindlimb function was tested in open field from 3 days post-injury (DPI) until the chronic phase at 154 DPI (Fig. 1a). In the acute phase, a spinal shock with predominantly left sided paraparesis occurred in all rats. Ipsilateral hindlimb function improved rapidly within the first 14 days in all animals. The BBB (Basso Beattie and Bresnahan) score vs. DPI curves of all animals showed a steep and almost identical course until 7 DPI. Overall, the functional improvement in the alginate group was better than in the control group that did not receive any treatment from 7 DPI on, and the gap between the curves progressively widened until 28 DPI. Afterward, we observed stagnation in both groups.

A significant effect of treatment was confirmed by two-way repeated measures of ANOVA (P < 0.001) and pairwise comparisons with Bonferroni post hoc *t* tests showed that the mean BBB scores of alginate-treated rats were significantly improved at several time points.

The frequency distribution of the BBB score at selected time points is depicted in Fig. 1b and approximates in most cases a normal distribution. Starting at 14 DPI, more alginate-treated rats achieved BBB scores ≥13, which marks the onset of frequent forelimb-hindlimb coordination. This is an important component of effective locomotion in rats and further evidence for better recovery of treated animals.

### Lesion dimension

**Figure 2:**
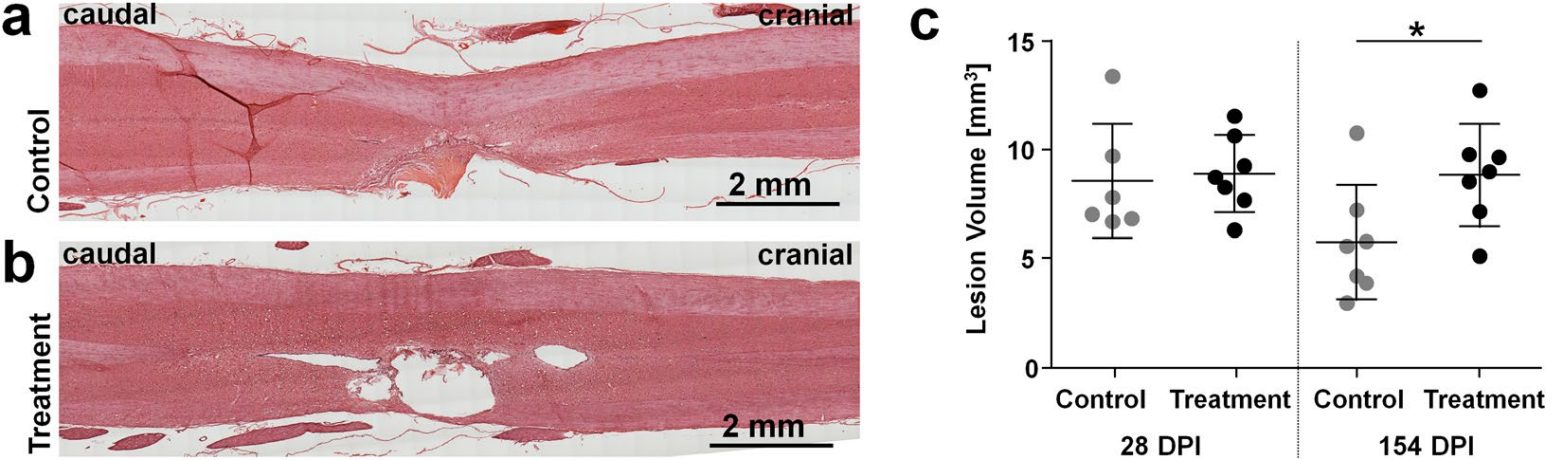
Morphology of the lesion. (**a**) Example of HE stained section of a sample of the control group at 154 DPI. (**b**) Example of HE stained section of a sample of the treatment group at 154 DPI. (**c**) Lesion volume, two-tailed t-test, *P = 0.04. The lesion volume included all injury-related changes visible using HE staining, namely scarring, interruption of continuity, and cyst formation.

The anatomical extent of the lesion and of tissue damage (micro-cysts) beyond the lesion was measured on hematoxylin-eosin (HE) stained sections (Fig. 2a,b). At 28 DPI, the lesion was larger than the nominal dimension of the hemimyelonectomy (∼6 mm^3^) in all animals, as shown in Fig. 2c. Treatment and control group did not display any significant difference. At 154 DPI, the lesion had shrunk below the nominal hemimyelonectomy dimension in a subset of control animals, while the mean lesion dimension of the treatment group did not change compared to the earlier time point. As a result, the mean lesion volume of the treatment group was significantly larger. However, animals with larger lesions did not show worse functional outcome; no negative correlation between lesion dimension and functions was found by analyzing the single animals, as illustrated in Supporting Fig. S1.

The larger lesion volume of the treatment group was related to the presence of larger cysts. The morphology of the contralateral spinal cord was generally better preserved in the treatment group, while collapsing of the contralateral white matter tract toward the lesion was more often observed in the control group (compare Fig. 2a,b).

### SCI visualized by label-free multiphoton microscopy

**Figure 3:**
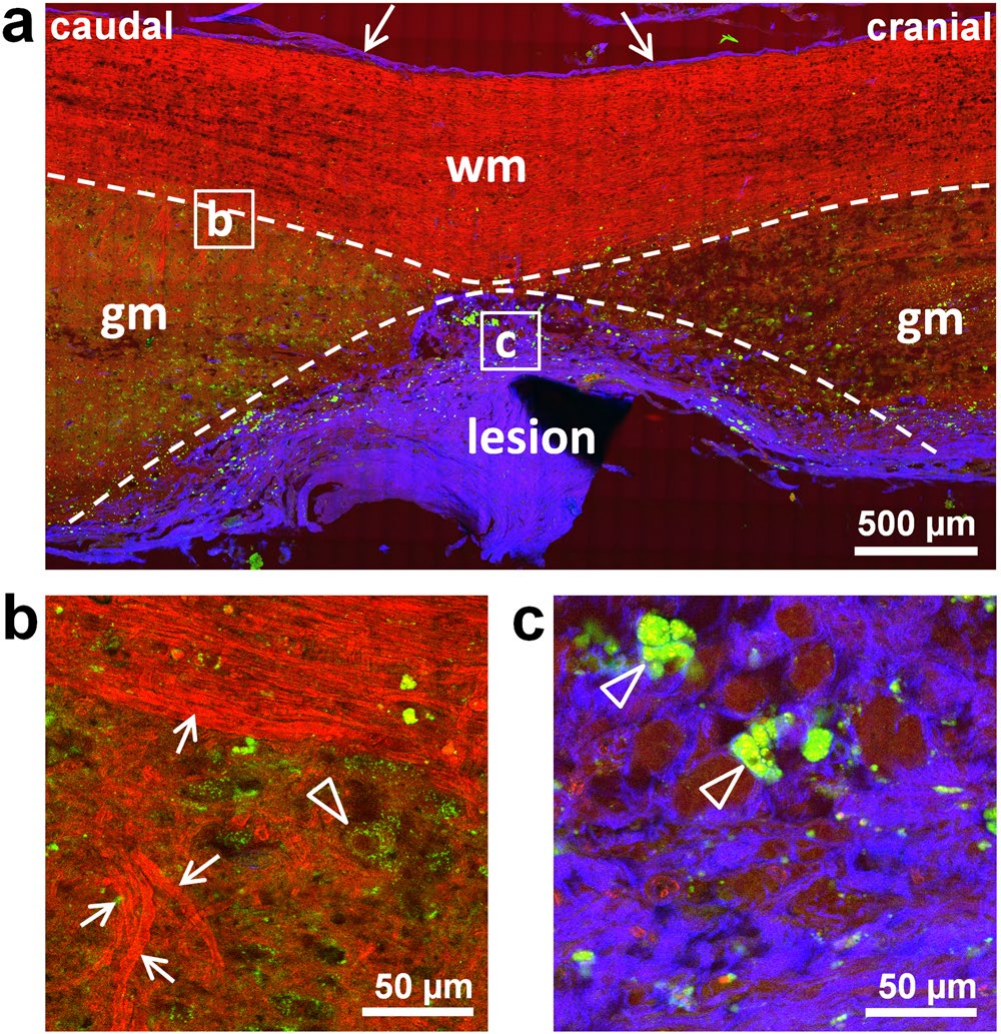
Spinal cord injury visualized by label-free multiphoton microscopy. RGB images obtained merging CARS (red), endogenous TPEF (green) and SHG (blue). (**a**) Longitudinal section of a sample of the control group at 154 DPI (HE staining of a consecutive section is shown in Fig. 2a); wm: white matter, gm: gray matter, arrows indicate the dura. (**b**) Magnification showing the tissue structure at the border between gray and white matter; arrows indicate myelinated axons, the arrowhead indicates a neuron. (**c**) Magnification showing the tissue structure in the lesion; arrowheads indicate inflammatory cells.

Figure 3a shows the structure of injured tissue and of surrounding spinal cord as visualized by label-free multiphoton microscopy of unstained rehydrated cryosection. CARS allows the identification of white matter and axons. Myelin sheaths are characterized by high CARS intensity and therefore myelinated axons, indicated by arrows in Fig. 3b, can be readily distinguished from other nervous structures. TPEF is generated by intrinsic cytoplasmic fluorophores such as NAD(P)H, and is particularly intense in activated inflammatory cells, both microglia and macrophages^33^. These cells were found especially inside or nearby the injury area (arrowheads in Fig. 3c). Moreover, large cells in gray matter were characterized by weak punctuate TPEF in the cytoplasm, and might represent neurons in agreement with previous findings^36^, or spinal cord astrocytes. An example of these is indicated by the arrowhead in Fig. 3b. SHG visualizes the dura, indicated by arrows in Fig. 3a, and the fibrotic scar at the lesion site.

Striking tissue alterations of spinal cord parenchyma, with massive accumulation of fluorescent inflammatory cells and dense collagenous scarring were found at the hemisection site and its immediate surroundings on the ipsilateral side, for a total extension not exceeding 4 mm. This focal tissue pathology is in agreement with other findings on spinal cord transection in rats^37^.

### Scarring

**Figure 4:**
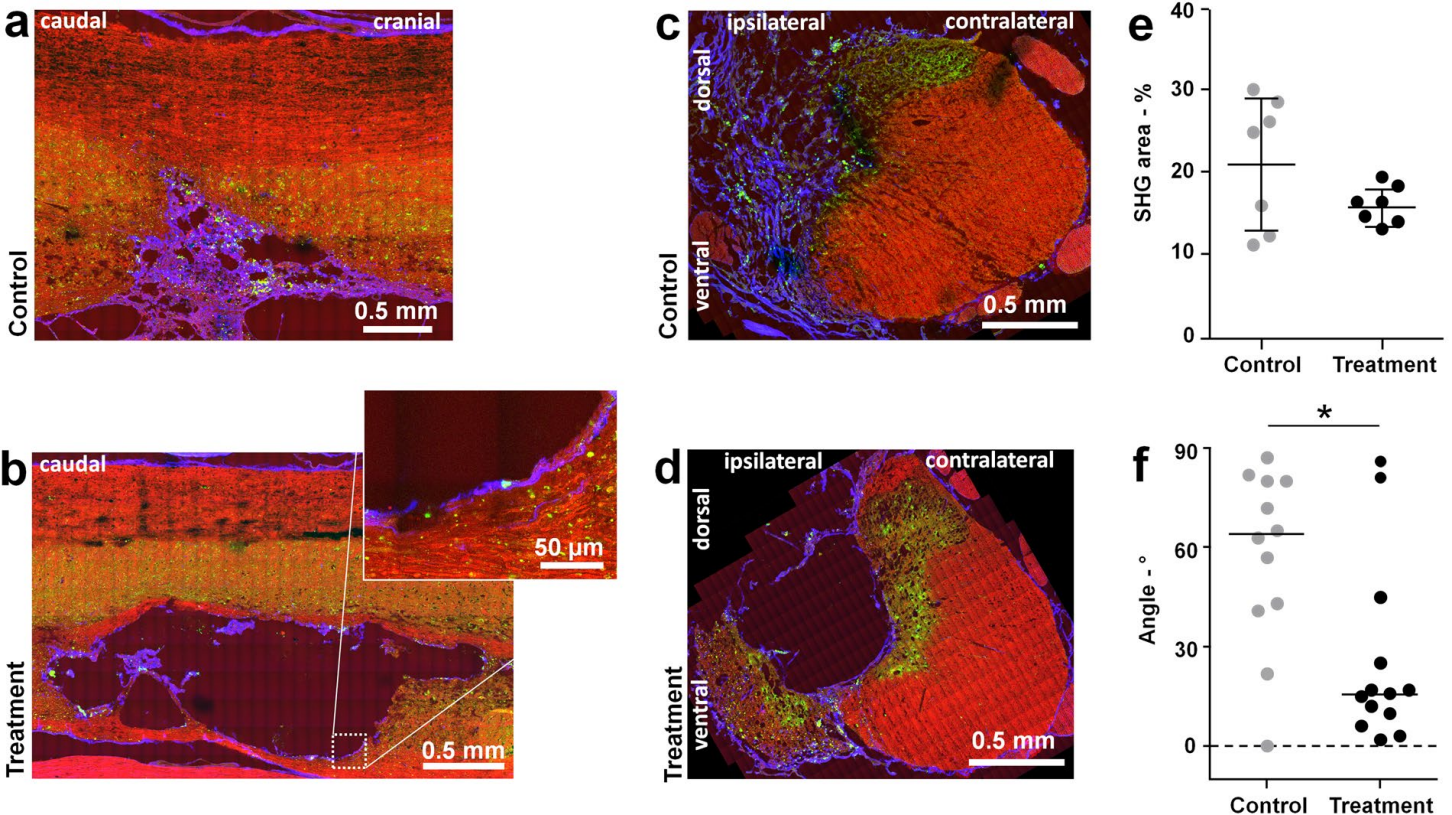
Fibrotic scarring. (**a**) Label-free multiphoton picture of a longitudinal section of a control spinal cord at 154 DPI. (**b**) Label-free multiphoton picture of a longitudinal section of a treatment sample at 154 DPI, with magnification of the collagenous lining of the cyst. (**c**) Label-free multiphoton picture of a cross section of a control sample at 154 DPI. (**d**) Label-free multiphoton picture of cross section of a treatment sample at 154 DPI. (**e**) Quantification of tissue area covered by SHG-active collagen on cross sections at 154 DPI (mean ± SD). (**f)** Evaluation of collagen fiber direction on longitudinal sections at 28 and 154 DPI; the median is indicated; Mann-Whitney test, *P = 0.021. CARS: red, TPEF: green, SHG: blue.

Collagenous scarring was evaluated using the SHG signal of the label-free multiphoton images. The scar tended to fill the lesion in the control samples, while a thin lining of collagen typically encapsulated the cysts in samples of treated animals. This observation applied at 28 DPI, as well as at 154 DPI as shown in Fig. 4a,b. Analysis of cross sections at 154 DPI confirmed the morphological difference of scarring between the two groups. While in the majority of samples of the control group a dense collagen meshwork filled the lesion (Fig. 4c), cysts with just a thin lining of collagen were mostly observed in the treatment group (Fig. 4d). Total amount of collagen in the tissue was quantified on cross sections as percent of area displaying a SHG signal and is shown in Fig. 4e. Massive deposition of collagen was observed in many samples of the control group, but in none of the treatment group. However, the difference between the mean content of collagen is not statistically significant.

The orientation of collagen fibers was evaluated on longitudinal sections and was found to be significantly affected by the treatment, irrespective from the time point, as illustrated in Fig. 4f. While in the control group the main direction of collagen fibers typically displays large angles to the longitudinal axis of spinal cord, longitudinal alignment is evident in the treatment group, as shown by clustering of the data below 30°.

The glial scar was addressed with GFAP immunofluorescence in combination with label-free multiphoton microscopy. The glial scar surrounding the fibrotic scar was observed in all animals, with large inter- and intra-animal variability in scar extension and astrocyte density at both time points. A reduction of the glial scar in treated animals could not be proven. A tendency of astrocytic processes to align with the spinal cord axis was found in a subset of treated animals at 154 DPI. Moreover, a thinner glial scar was most often observed in combination with a thin fibrotic scar typically surrounding cystic spaces, as it can be seen in Supporting Fig. S2.

### Myelin degradation and axonal demyelination

**Figure 5:**
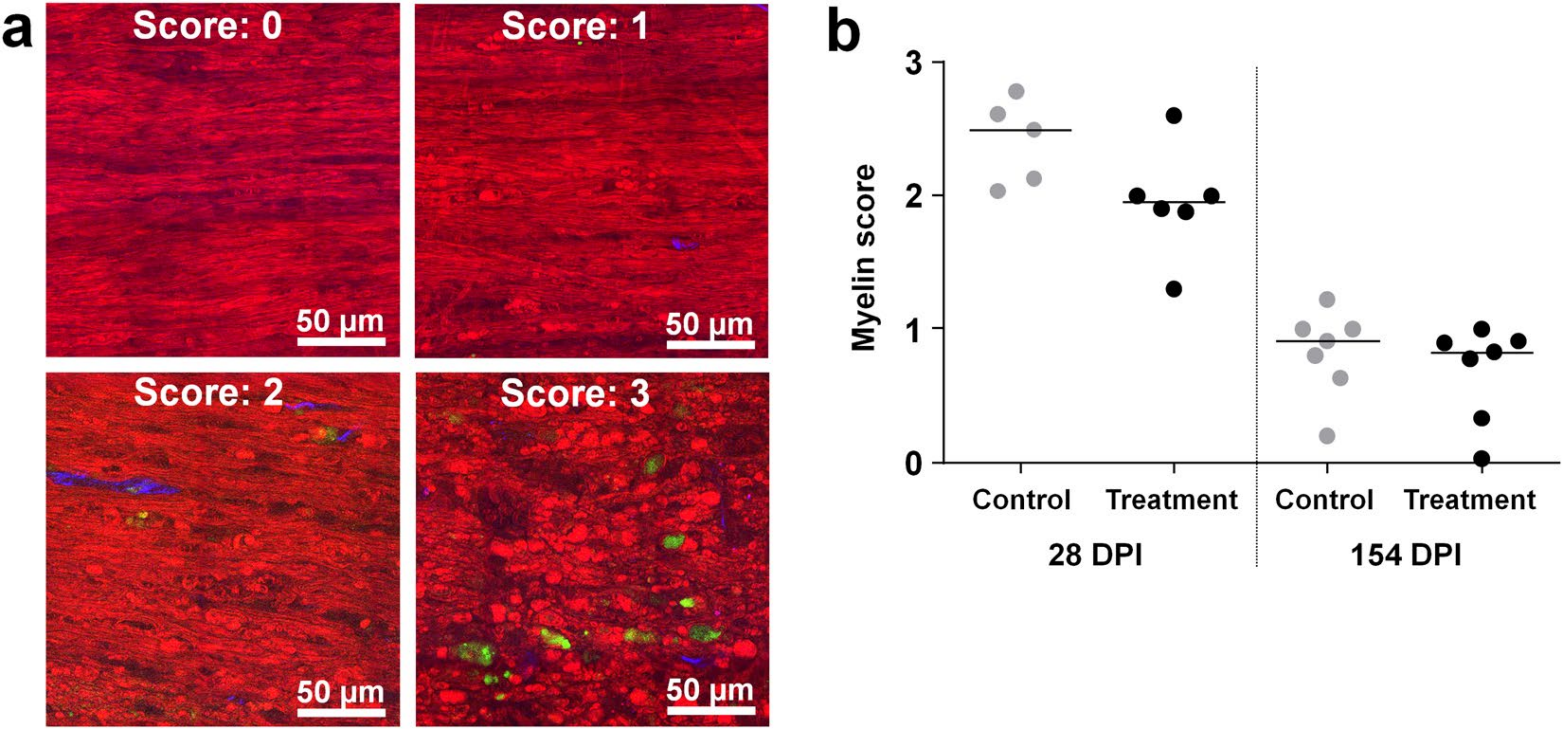
Myelin morphology. (**a**) Different myelin morphology in label-free multiphoton pictures and associated scores. Score “zero”: normal white matter; scores 1 and 2: increasing presence of lipid droplets and disturbed axonal alignment; score 3: predominant lipid droplets and loss of any recognizable axonal alignment. CARS: red, TPEF: green, SHG: blue. (**b**) Myelin scores (the median is indicated).

Myelin degradation inside and around the injury at 28 and 154 DPI was evaluated using the CARS signal of images of longitudinal spinal cord sections. The morphology of white matter was analyzed based on presence of ovoidal myelin fragments and lipid droplets. Myelin ovoids visualized by label-free chemical imaging have been shown earlier to be unequivocally associated with nerve degeneration^38^. Scores from zero to three were associated with increasing levels of degradation based on presence of ovoidal myelin fragments and the degree of axonal alignment, as shown in Fig. 5a. Most of the animals at 28 DPI displayed scores between two and three, indicating strongly altered white matter morphology with myelin degradation (Fig. 5b). At 154 DPI, myelin degradation is resolved and residual lipid droplets were observed only in a few regions. All animals had scores close to or lower than one. The implant did not have any significant influence on myelin morphology at either time point.

**Figure 6:**
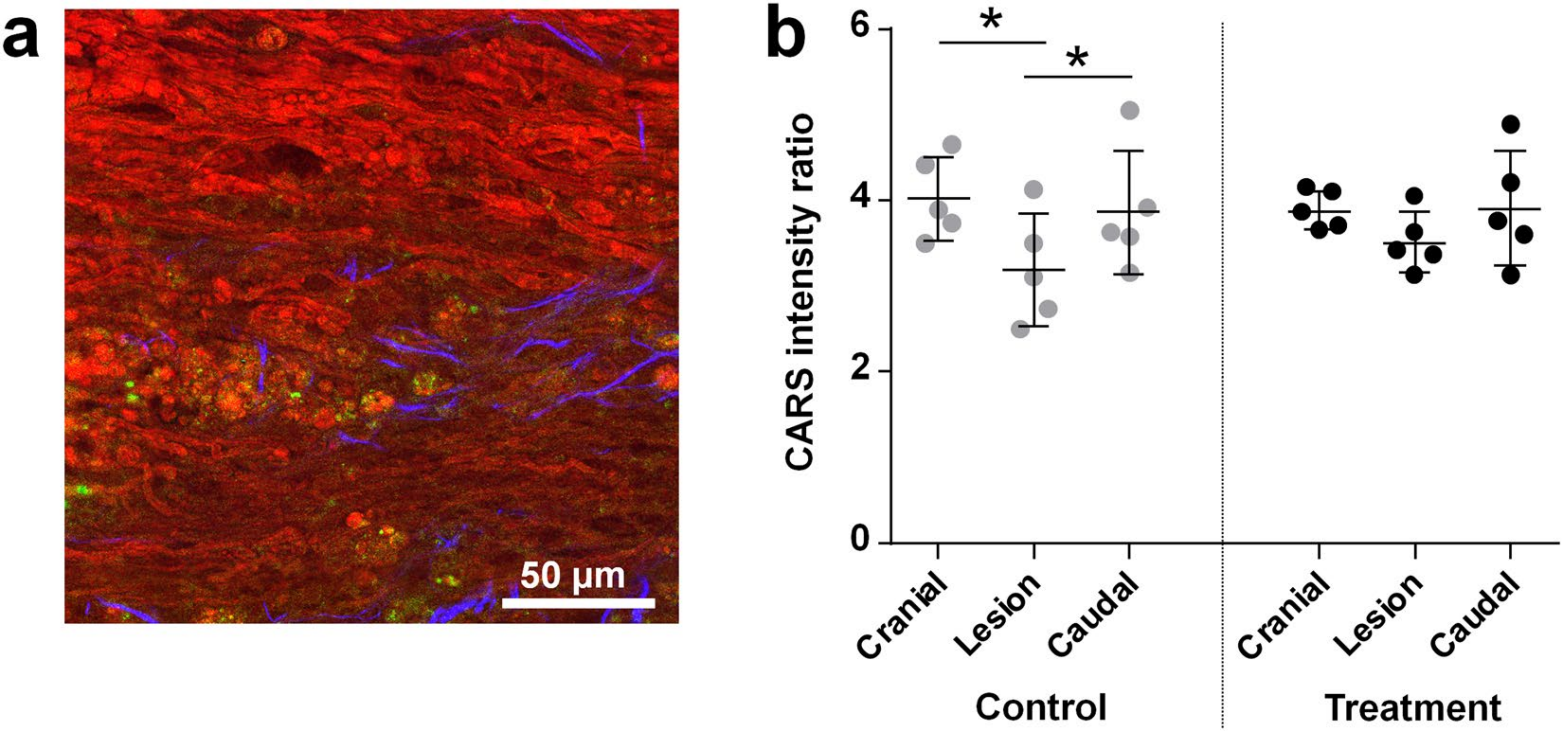
Axonal demyelination of contralateral white matter. (**a**) Demyelination observed at 28 DPI by label-free multiphoton microscopy of a longitudinal spinal cord section of a treated animal; myelinated axons are visible in the upper part, demyelinated ones in the lower part; CARS: red, TPEF: green, SHG: blue. (**b**) CARS signal intensity in the contralateral white matter measured on cross sections at 154 DPI (mean ± SD, one-way repeated ANOVA, *P < 0.05).

White matter regions, in which axonal structures are visible, but exhibit much lower CARS signal, were interpreted as demyelination, as shown in Fig. 6a. Axonal demyelination was clearly observed on longitudinal sections at 28 DPI in the ipsilateral white matter close to the lesion and, in few cases, also in the contralateral white matter. At 154 DPI, only control animals were characterized by significant demyelination of the white matter contralateral to the lesion, as revealed by analysis of CARS signal intensity on spinal cord cross-sections (Fig. 6b).

### Axons within the lesion

**Figure 7:**
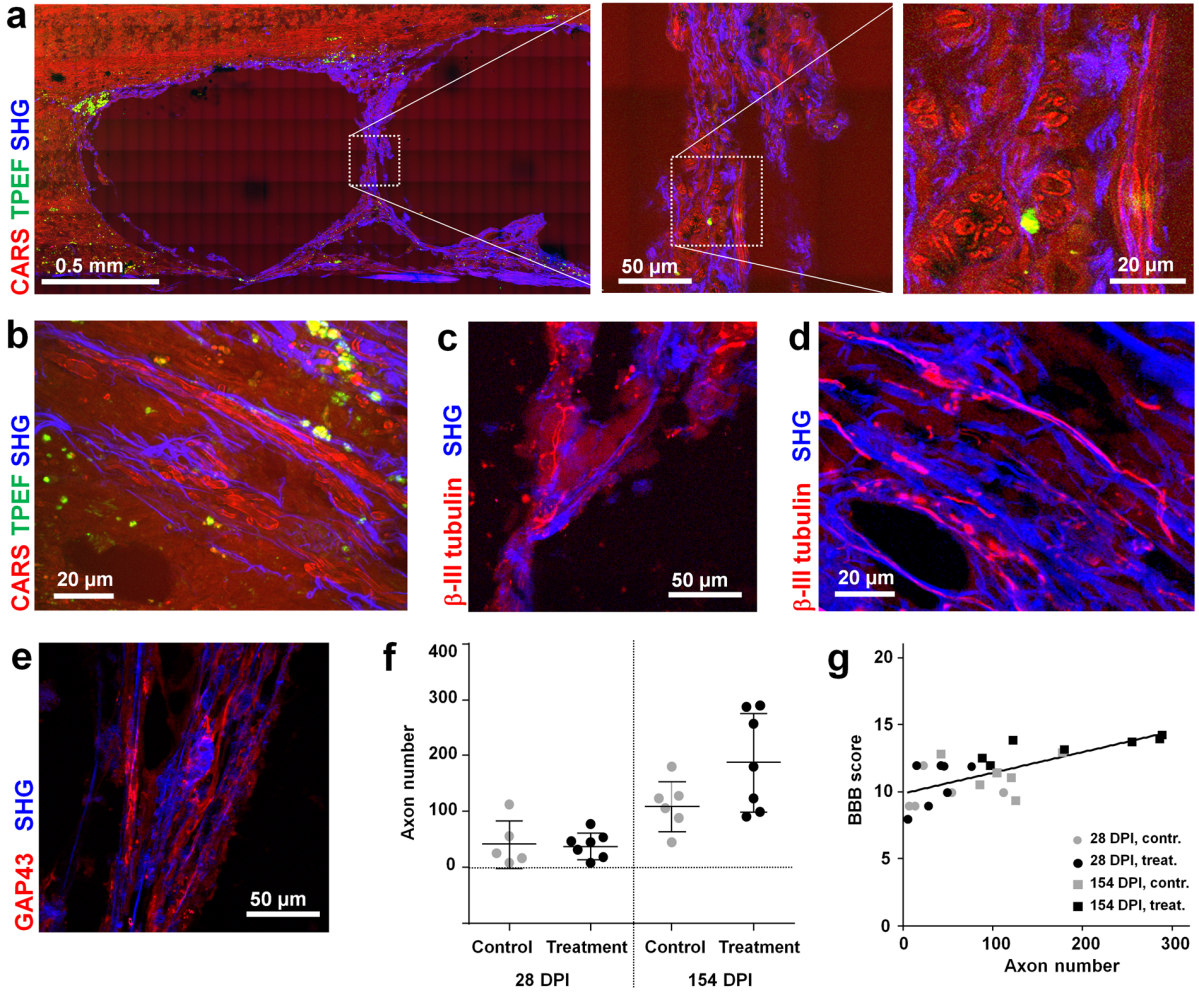
Axons in the lesion. (**a**,**b**) Label-free multiphoton pictures of longitudinal sections of samples of the treatment group at 154 DPI; CARS: red, TPEF: green, SHG: blue. Axonal structures are clearly visualized by CARS at high magnification. (**c**) Immunohistochemistry of a non-consecutive section of the sample shown in (**a**) visualized by multiphoton microscopy (red: TPEF of β-III tubulin, blue: SHG). (**d**) Immunohistochemistry of a non-consecutive section of the sample shown in (b), visualized by multiphoton microscopy (red: TPEF of β-III tubulin, blue: SHG). (**e**) GAP43 immunohistochemistry for growing axons of a non-consecutive section of the sample shown in (**a**) visualized by multiphoton microscopy (red: TPEF of GAP43, blue: SHG). (**f**) Axon counts within the lesion (mean ± SD). (**g**) Scatter plot of functions vs. axon counts. BBB scores indicated as 154 DPI are the average of scores obtained for each rat between 70 and 154 DPI.

The presence of myelinated axons in the lesion was evaluated using the CARS signal of images of longitudinal sections. Axons were considered to be localized in the injured area when concurrent presence of collagen was confirmed by the SHG signal. At 28 DPI, few scattered axons were detected in the external parts of the lesion of both treatment and control groups. At 154 DPI, axons were visible inside the scarring tissue in both experimental groups, as well as in the thin bridges of tissue crossing the cysts in the samples of the treatment group, as shown in Fig. 7a. The axons and the collagen fibers in the lesion were often parallel, as in the example of Fig. 7b. Immunohistochemistry confirmed both observations, showing the presence of β-III tubulin positive structures in the tissue bridges (Fig. 7c) as well as their alignment with collagen fibers (Fig. 7d). Moreover, GAP 43 immunohistochemistry demonstrated the presence of sprouting axons in the tissue bridges crossing the cysts (Fig. 7e) and in the tissue surrounding the cysts in animals of the treatment group.

At 28 DPI, the axon number evaluated on CARS images was lower and there was no implant effect (Fig. 7f). At 154 DPI, three treated animals were characterized by very high axon counts, although the overall difference between groups was statistically not significant. Finally, the BBB score of each animal were plotted versus axon count and a linear regression was performed (r^2^ = 0.46) with positive slope significantly different from zero (P < 0.001), as shown in Fig. 7g. This indicates a correlation between axons and better functional performances.

### Inflammation

**Figure 8:**
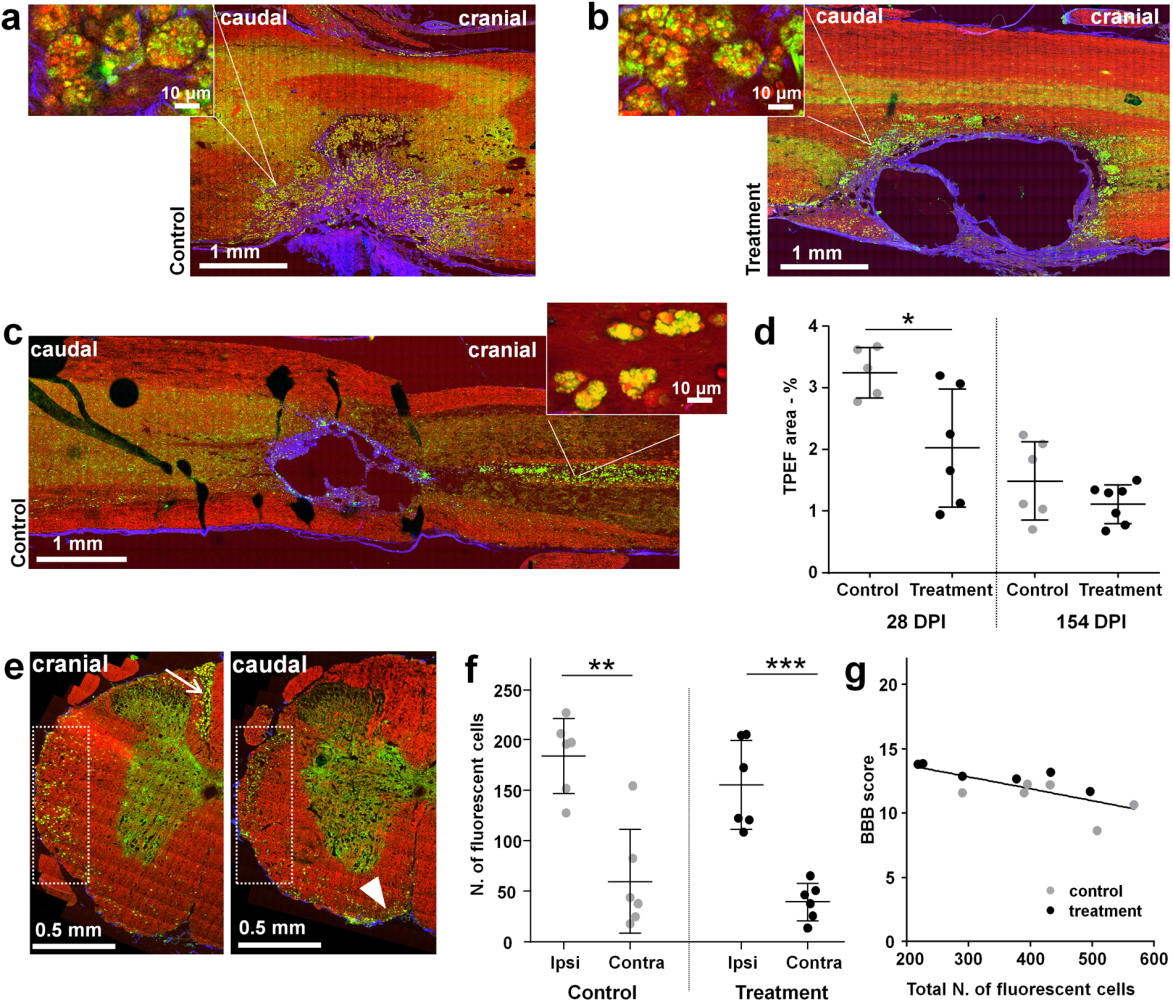
Inflammation. (**a**) Label-free multiphoton picture of a longitudinal section of a control sample at 28 DPI. (**b**) Label-free multiphoton picture of a longitudinal section of a treatment sample at 28 DPI. Magnifications show inflammatory cells. (**c**) Label-free multiphoton pictures of a longitudinal section of a control sample at 154 DPI, with magnification of the inflammatory cells in the dorsal median sulcus. CARS: red, TPEF: green, SHG: blue. (**d**) Quantification of area covered by fluorescent inflammatory cells in the injured area of longitudinal sections (mean ± SD, two-tailed t-test *P < 0.05). (**e**) Label-free multiphoton pictures of ipsilateral side of cross-sections located cranially and caudally to the lesion of a control sample at 154 DPI; the arrow indicates fluorescent inflammatory cells accumulating cranially in the dorsal median sulcus, and the arrowhead caudally near the ventral fissure; boxes indicate a region with presence of scattered inflammatory cells. (**f**) Quantification of fluorescent inflammatory cells in the ipsi- and contralateral white matter on cross-sections at 154 DPI (mean ± SD, two-tailed t-test, **P < 0.01, ***P < 0.001). (**g**) Scatter plot of ipsilateral hindlimb functions vs. number of fluorescent inflammatory cells. BBB scores are the average of scores obtained for each animal between 70 and 154 DPI.

Inflammatory cells were evaluated using the TPEF signal of label-free multiphoton images, based on methods validated elsewhere^33^. Additionally, matching between cells displaying strong endogenous TPEF and activated microglia/macrophages was verified by comparison with Iba1 immunohistochemisty (Supporting Fig. S3). At 28 DPI, fluorescent inflammatory cells accumulate in the resection cavity of control samples as shown in Fig. 8a, or around the implant in treatment samples as shown in Fig. 8b. Fluorescent cells at this time point are laden with lipid droplets and are consistent with foam cells (magnifications in Fig. 8a,b). At 154 DPI, fewer fluorescent cells were observed in the lesion of control samples, as shown in Fig. 8c, as well as around the implant in treated samples. Quantitative analyses confirmed that the inflammation was significantly higher at 28 DPI compared to 154 DPI (Fig. 8d). The samples of treated animals displayed lower inflammation, and the difference compared to controls was significant at 28 DPI. A weak degree of negative correlation between extent of inflammation at the lesion site and hindlimb function was found (Supporting Fig. S4).

Outside the lesion, fluorescent immune cells consistent with foam cells accumulated in the dorsal median sulcus cranial to the lesion at 154 DPI, irrespective of the treatment (Fig. 8c). Additionally, scattered immune cells were detected in ipsilateral and contralateral white matter some millimeters away from the lesion, where the tissue morphology appears normal in all other aspects.

TPEF images of cross-sections located ∼4 mm away from the lesion were analyzed in order to refine the evaluation of this diffuse type of chronic inflammation, confirming high immune cell occurrence in the dorsal median sulcus cranial to the lesion (arrow in Fig. 8e) and sparse immune cells in the white matter, both cranial and caudal to the lesion (boxes in Fig. 8e). Furthermore, the analysis revealed many cells near the ventral fissure on the caudal sections in all samples (arrowhead in Fig. 8e).

The quantification of immune cells number in the white matter cranial and caudal to the lesion revealed a significantly higher number of cells in the ipsilateral white matter, as shown in Fig. 8f. Although the inflammatory cells were generally less in the treated samples (see also Supporting Fig. S5), the effect of implants was not statistically significant. However, a relationship exists between hindlimb functions and inflammation in the chronic phase, as revealed by the scatter plot of BBB score vs. total number of inflammatory cells on both spinal cord sides shown in Fig. 8g. The linear regression indicates a correlation (r^2^ = 0.52) with negative slope significantly different from zero (P < 0.01).

## Discussion

Up to date, there is no approved clinical regenerative therapy for spinal cord injury. Animal studies investigated mostly combined approaches incorporating stem cells, neurotrophic factors and 3D matrix/scaffolds. Besides the specific nuances in “readiness” to regenerate of species, a major difficulty in translating any therapeutic approach from the laboratory into clinical studies is the predictability of its adverse effects. Alginate hydrogels offer a major advantage in this context, since they are already established as biocompatible inert materials in the clinical context^39,40^. Easy handling and modifiability prior to implantation renders them suitable for spinal cord laceration injuries.

Therefore, we investigated functional and morpho-chemical effects of non-functionalized soft alginate implants in a thoracic hemisection model in rats. We found the following implant- related effects: 1) function improvement starting already in the sub-acute phase; 2) thinner collagen scar, with collagen fibers more often longitudinally oriented; 3) increased number of myelinated axons in the lesion; 4) decreased contralateral demyelination; 5) reduced accumulation of inflammatory cells at the lesion; 6) decreased chronic inflammation.

Treated rats showed BBB scores that are on average two scores higher than those of control animals in the chronic phase. The mean BBB improvement after interventions with functionalized biomaterials in hemisection models retrieved in a systematic review^41^ is moderate as well (4.44, 95% CI = 2.65–6.24), considering the use of neurotrophic factors and cell grafts in those studies. Overall, we observed that the beneficial effects of alginate implantation on hindlimb function were swifter and more pronounced until 14 DPI. Spontaneous locomotor improvement observed in the first two weeks post injury has been attributed to recovery from initial spinal shock and enhanced plasticity of the spared nervous tissue^42,43^. Treated animals performed significantly better compared to controls already in this early phase, suggesting that alginate intervenes already in the acute phase following SCI.

The presence of alginate implants strongly altered the morphology of the nervous tissue inside and around the injury. Alginate implants were associated with presence of larger cysts, but these had no adverse effects on hindlimb functions. Rats underwent an anatomically complete disconnection between cranial and caudal parts of the ipsilateral side of spinal cord. All descending tracts important for gait (corticospinal, rubrospinal and descending serotonin pathways) were interrupted for a length of 2 mm. In both control and treatment group, we observed scarring or presence of cysts in the injured hemicord involving tissue until the midline in the chronic stage after SCI (as can be seen in Fig. 4). Therefore, the presence and localization of cysts was not considered to be substantial for functional outcome of the ipsilateral hindlimb. In earlier studies, it was shown by using both stainings and vibrational spectroscopy^24,25^, that the alginate hydrogel used in this study is not fully degraded and remains in the cysts up to six months after implantation. We verified that the presence of the implant influenced fibrous scar morphology. The alginate-filled cysts were encapsulated by a layer of collagen that was thin and sometimes discontinuous. This observation confirmed earlier results obtained with non-functionalized calcium alginate hydrogel implants^25,44^. The glial scar surrounded the fibrous scar in all samples and was not altered by the treatment.

The hydrogel had positive effects on the amount of myelinated axons that were detected by CARS at the injury site, although massive ingrowth of axons in the hydrogel could not be demonstrated in agreement with earlier studies^44^. As collagen infiltrated also in the tissue surrounding the injury, it was not possible to discern by label-free multiphoton microscopy between spared axons at the border of injury and axons growing into the lesion. However, the long and thin tissue bridges crossing the large cystic cavities (observed in 5/7 treated animals both at 28 and 154 DPI) represent new tissue infiltrating within cracks of the implants. Axons within this tissue bridges represent axons that regrew after injury, as also confirmed by immunohistochemistry. In addition, the alignment observed between axons and collagen fibers in the lesion further support the idea that these are regrowing axons. Moreover, the correlation between axons in the lesion and hindlimb functions may indicate that at least a subpopulation of these axons had reconnected.

The data show that the orientation of collagen fibers was affected by alginate implantation: the fibers displayed a higher degree of longitudinal alignment in the major part of the samples of the treatment group. Axons in the lesion were mostly aligned with collagen fibers. Hence, the collagen meshwork might have worked as a guiding structure that helped axons to bridge the injury. Although the dense fibrotic scar that forms in the lesion after SCI serves as a binding matrix for several inhibitory molecules and is recognized as major impediment for axonal regeneration^45–47^, collagen type I does not inhibit axonal growth “per se”. Indeed, it was shown that collagen implants support the ingrowth of injured fibers in rat models of SCI^48,49^, and that properly oriented collagen fibers can act as scaffold for guiding axons across the lesion^50^.

Further beneficial effects of alginate hydrogel implants were observed on contralateral white matter demyelination. While myelin degradation of the ipsilateral white matter proceeded unaffected by the treatment in the intermediate phase, the level of demyelination in the contralateral white matter at the chronic stage was reduced in presence of implants. As demyelination was not observed one-month post injury in similarly treated rat models^25^, we hypothesize that the higher degree of axonal myelination of contralateral white matter is not attributed to remyelination, but rather to neuroprotective effects of alginate implants. Other studies evidenced contralateral white matter demyelination in the chronic injury phase after dorsal hemisection in rats^51^. The time point, when the decline of transmission through the contralateral white matter reached a maximum, marked also the end of locomotor recovery^51^, indicating that contralateral demyelination plays an important role in functional outcome.

Hydrogel implantation did not exacerbate the inflammatory response, in agreement with the generally accepted biocompatibility of commercial-grade (i.e., high purity) alginate^14^. The results of this study rather suggest decreased inflammation. The implant prevented the accumulation of debris and infiltration of cells in the resection cavity, leading to a significantly lower amount of activated microglia and macrophages during the intermediate phase. Similar distribution of inflammatory cells in control and alginate hydrogel implanted rats was observed in earlier studies^44^. The reduced inflammation might indicate reduced secondary damage, which correlates with the swifter functional improvement observed in treated rats during the acute and sub-acute phases.

Residual inflammation was observed until a late injury phase, in accordance with earlier studies. Chronic alterations of spinal cord white matter after contusion injury in rats were observed 4 mm rostral and caudal to the injury epicenter 6 weeks after SCI, and included increased microglia/macrophages in the dorsal, lateral and ventromedial regions^52^. Chronic inflammation with persistency of macrophages with foamy phenotype was observed in rats until 55 DPI^53^. Our results show that a similar distribution of activated cells with a phenotype consistent with foam cells remains also in a later stage of unresolved inflammation. Moreover, lower levels of inflammation outside the lesion in the chronic phase positively correlated with the improved functions. The role of inflammation is crucial for outcome after SCI^54^. Ongoing inflammation in the intermediate and chronic phase of SCI in rodents is dominated by neurotoxic M1-type macrophages^55^, which localize both at the lesion epicenter and in the area of axonal degeneration^56^, and was demonstrated to be highly detrimental for recovery^57^.

By using label-free multiphoton microscopy, we could simultaneously address three main issues after SCI: (i) presence and myelination of axons, (ii) amount and morphology of fibrotic scarring (iii) number and localization of inflammatory cells. A subset of morphochemical parameters visualized by multiphoton microscopy correlated with locomotor functions. Moreover, the intrinsic lack of tissue processing of our label-free approach enabled imaging of the fragile and tiny tissue bridges extending in the cystic cavities. The main limitation of the label-free multiphoton microscopy modalities used here is the incapability to address the glial scar. However, other similar techniques could fill this gap, such as stimulated Raman scattering, which enables visualization of astrocytic processes based on selective chemical imaging of proteins^58^.

In conclusion, we analyzed the effects of soft calcium alginate hydrogel in a rat model of hemisection, and found that, even without any functionalization, the implants positively affected the sequence of events after injury. Implant effects were primarily neuroprotective: contralateral demyelination and inflammation were reduced. The alginate hydrogel implants likely had also regeneration-promoting effects: the amount of myelinated and probably functional axons was increased in the lesion and clearly correlated with improved functions. Finally, the morphology of the fibrotic scar was deeply affected, possibly contributing to an environment that facilitated axonal bridging across the injury. Overall, we conclude that alginate hydrogel promotes a regeneration friendly environment after a massive spinal cord injury.

## Methods

### Ethics statement and animal care

All animal experiments were performed in accordance with the guidelines of the TU Dresden, based on national laws in full agreement with the European Union directive on animal experiments. They were approved by the Regional Council (Landesdirektion Sachsen, Germany, AZ 24–9168.11-1/2013-37). All efforts were made to minimize animal suffering. The analgesic carprofen (5 mg/kg; Veicore Ltd, UK) and a prophylactic antibiotic (Duphamox LA 15 mg/kg; Fort Dodge Veterinär GmbH, Germany) were given preoperatively. Surgery was performed on rats anesthetized with an intraperitoneal injection of xylazine/ketamine (10 mg/kg xylazine; 90 mg/kg ketamine). In the first two postoperative days, analgesia was achieved with carprofen (5 mg/kg s.c.) once a day subcutaneously. Rarely, buprenorphine (0.05 mg/kg s.c.) was given for additional analgesia. Injured animals received daily care.

### Alginate hydrogel

Pronova ultrapure sodium alginate (Novamatrix, Sandvika, Norway), containing 50% mannuronate, was used to prepare the hydrogel. Sterile filtrated 4% alginate solution was produced. The alginate hydrogel was prepared by overlaying the sol with a crosslinking solution of CaCl2 and NaCl for 12 h at room temperature. The shear storage modulus of the obtained hydrogel was G’ = 0.195 kPa. Further details about preparation and rheology are reported elsewhere^25^.

### Animal experiments

Female Wistar rats (n = 42), aged 16 weeks and weighting between 200 g and 250 g, were used. A left-sided hemisection of the spinal cord at the level T9-T10 of the thoracic vertebrae was surgically induced under a surgical microscope. The left side of the spinal cord was dissected along a medial longitudinal plane for a length of 2 mm as described earlier^25,59^. Randomly selected rats (n = 21) received a soft calcium alginate hydrogel into the lesion. The blocks of alginate hydrogel with dimension approximately 2 mm × 2 mm × 1.5 mm (medio-lateral × rostro-caudal × dorso-ventral, corresponding to a volume of ∼6 mm^3^) were manually cut and placed in the hemisection immediately after injury induction. The remaining rats (n = 21) did not receive any implant and were used as control. The rats were sacrificed at 28 days post injury - DPI (treatment group: n = 7, control group: n = 7) or at 154 DPI (treatment group: n = 14, control group: n = 14). The animals were perfusion-fixed using 4% paraformaldehyde in TRIS-buffered saline, the spinal cords were isolated and cryoprotected in rising sucrose concentration (10% for 24 h and 30% for 24 h). A ∼20 mm long portion of the spinal cord with the lesion roughly in the center was embedded in tissue freezing medium (Leica, Nussloch, Germany) and frozen on dry ice. Finally, 16 μm thick cryosections were prepared. Longitudinal sections were obtained from all rats sacrificed at 28 DPI and half of the rats at 154 DPI (treatment group: n = 7, control group: n = 7); transverse cross-sections were obtained from the remaining rats at 154 DPI (treatment group: n = 7, control group: n = 7). Cryosections were stored at −20 °C until use.

### Behavioral tests

Beginning 10 days prior to surgery, all rats were trained twice daily to facilitate the accommodation to the open field. After surgery, hindlimb functionality was assessed in open field at 3, 7, 14, 21, 28 DPI and afterwards every 14 days until 154 DPI. The rats were placed for 4 minutes onto the center of an area 100 cm × 100 cm large with smooth non-slippery surface, and the locomotor functions assessed using the Basso, Beattie and Bresnahan (BBB) rating scale^60^. The scores were determined as an average of two observers and blind scoring ensured that observers were not aware of the animal’s treatment. A third observer that was not blinded filmed the session. In case of deviating scores given by the two observers, the video was analyzed and the best achieved BBB score was assigned.

### Histology

The sections were fixed in methanol–acetone (1:1) for 10 min at −20 °C.

For HE staining, the sections were washed in distilled water, and incubated in Meyer’s hematoxylin/hemalum for 3 min. After washing in distilled water, the tissue was briefly destained in HCl–ethanol solution. Washing using tap water for 5 min was followed by 3 min staining in eosin (1% eosin G in 80% ethanol). The sections were dehydrated with rising ethanol concentrations, cleared in xylene and coverslipped using DePex.

### Immunohistochemistry

Heat antigen retrieval in citrate buffer was performed followed by 0.3% TritonX for 15 min and blocking in 5% bovine serum albumin in PBS for 1 h. For GFAP immunohistochemistry, the tissue was probed with the antibody (Anti-GFAP-Cy3, 1:200, Sigma Aldrich) overnight at 4 °C. Rabbit anti- β-III-tubulin (1:500, Covance Inc., Princeton, NJ, USA), rabbit anti-Iba1 (1:200, Wako Chemicals GmbH, Neuss, Germany) and rabbit anti-GAP43 antibody (1:250, abcam, Cambridge, UK) were incubated overnight at 4 °C. After washing with PBS, sections were probed with fluorescent secondary antibodies (donkey anti-rabbit Alexa Fluor 594 (1:500, Invitrogen, Thermo Fisher Scientific, Waltham, MA, USA) at room temperature for 1 h. Stained fluorescent structures were visualized using a microscope Axio Examiner Z.1 equipped with camera AxioCam and metal halide lamp HXP 120c (Carl Zeiss AG, Jena, Germany), or by two-photon fluorescence using multiphoton microscopy.

### Measurement of the lesion volume

The volume of lesions was evaluated on image stacks of serial longitudinal HE stained sections using a Stereo Investigator Software module (MBF Bioscience, Williston, USA) on a microscope Axioplan 2 (Carl Zeiss Microscopy GmbH, Jena, Germany). One section every 160 µm was used to create the stack (approx. 20 to 25 sections for each animal). The lesion volume was estimated using the Cavalieri method with grid size of 150 µm to measure the lesion area on each section. The lesion area was defined including the (cystic) cavities and surrounding scar. The efficiency of sampling was checked by estimation of coefficients of error, which varied between 0.017 and 0.041. Measurements were performed in a blinded fashion.

### Multiphoton microscopy

The system was already described elsewhere^61^. Briefly, an upright microscope Axio Examiner Z.1 was coupled to a laser scanning module LSM 7 (Carl Zeiss Microscopy GmbH, Jena, Germany) and controlled by the software ZEN 2010, Rel. 6.0. Two picosecond lasers (Femto Fiber pro NIR and TNIR, Toptica Photonics AG, Gräfelfing, Germany) emitting at 781 nm and 1005 nm respectively were used to simultaneously excite the SHG and TPEF signals, as well as the CARS signal of the symmetric stretching vibration of methylene groups (i.e., Raman band at 2850 cm^–1^). A W Plan-Apochromat 20×/1.0 objective was used. SHG and CARS were detected in transmission mode using 390 ± 9 nm and 640 ± 7 nm bandpass filters, respectively. Green endogenous TPEF was detected in reflection mode in the range 525 ± 25 nm, while red TPEF of Cy3 and Alexa Fluor 594 of immunohistochemical stained sections was acquired in the range 587.2 ± 22.5 nm. The single channel intensity images were acquired with 8 bit depth and combined as RGB images for presentation. The color coding of label-free images is the following: blue channel: SHG, green channel: endogenous TPEF, red channel: CARS. The color coding of images of labelled sections is the following: blue channel: SHG, green channel: endogenous TPEF, red channel: TPEF of Cy3 or Alexa Fluor 594. A tiling procedure provided by ZEN was used for acquisition of images larger than the field of view. Pixel dimension was set to 0.1 µm for single field of view images, to 0.2 µm for large stitched images of longitudinal sections, and to 0.3 µm for large stitched images of cross-sections. For label-free multiphoton imaging, the cryosections were rehydrated with PBS and coverslipped.

### Analysis of multiphoton images

All image analysis was performed in Fiji^62^.

Fibrotic scarring was analyzed based on collagen fibers imaged by SHG. Single-channel SHG images of longitudinal sections were manually segmented to select the injured area and excluding the dura. Collagen fiber orientation in the lesion was retrieved using the Directionality tool of Fiji with local gradient orientation method. For each sample, the direction displayed by highest number of fibers was chosen and reported as absolute value of the angle to spinal cord longitudinal axis. The amount of fibrotic scarring was evaluated based on area covered by collagen fibers. SHG images of cross sections were manually segmented to include all spinal cord tissue, but excluding cystic spaces. Afterwards, intensities were adjusted using the auto min/max tool, and the percent area with the signal of interest was selected with triangle threshold filter. The value for each animal was obtained by averaging the area retrieved on two sections inside the lesion 1 mm apart from each other.

Evaluation of myelin morphology was performed by visual inspection of CARS images of longitudinal sections, using 12 fields of views of dimension 200 µm × 200 µm acquired in the tissue immediately around the lesion. Myelin morphology in each field of view was rated with a score from zero to three, corresponding to increasing levels of damage as imaged by CARS (0: normal white matter morphology with well aligned axons, 1: sporadic presence of myelin ovoidal fragments within axons; 2: massive presence of myelin ovoidal fragments and lipid droplets, with disturbed axonal alignment; 3: predominant lipid droplets and loss of axonal alignment). The scores were then averaged for each animal.

Axons inside the lesion were identified based on morphology shown by CARS images and manually counted in 12 fields of view of dimension 200 µm × 200 µm in the lesion region.

Quantification of contralateral white matter demyelination was performed on CARS images of cross sections, in a region of 240 × 450 μm^2^ in the close proximity to the gray matter on the contralateral side to the lesion. The intensity of the CARS signal was adjusted using the auto min/max tool and CARS intensity threshold was then determined using the triangle filter in order to select the myelin sheaths only. Based on this, a mask was created and applied onto the original CARS image. The CARS signal intensity was measured in the selected regions only, thus excluding the background signal coming from intra- and inter-axonal spaces devoid of myelin. The intensity of the CARS signal was measured and ratioed to the background CARS signal of each image individually, in order to correct for small variations of excitation power and collection efficiency. The quantification was performed on: i) two sections 1 mm apart both located inside the lesion, ii) two sections located 2 mm and 4 mm away from the lesion, both cranially and caudally. The values obtained inside the lesion as well as cranially and caudally to the lesion were then averaged.

Inflammation at the lesion site was quantified based on the area covered by fluorescent cells imaged by TPEF. Single-channel TPEF images of unstained longitudinal sections were manually segmented to select a region of the ipsilateral tissue roughly 4 mm long, including the lesion site and altered surrounding tissue, but excluding cystic spaces. Afterwards, intensities were adjusted using the auto min/max tool and the percent area with the signal of interest was selected with maximum entropy threshold filter, which allowed to select highly fluorescent structures consistent with activated immune cells excluding other low-fluorescent cells^33^.

### Statistics

Statistics was performed using GraphPad Prism 6.05 (GraphPad Software, La Jolla, California, USA). Repeated measures one- or two-way ANOVA and Bonferroni post hoc t-test, two-tailed t-test or Mann-Whitney U-test were used. Results were considered statistically significant if P < 0.05. All statistics on data extracted from multiphoton images was performed on groups formed by 5 to 7 rats (being 7 the number of animals of each experimental group - see section “Animal Experiments”), as in some cases it was not possible to obtain data due to problems in tissue handling.

### Data availability

The datasets generated and analyzed during the current study are available from the corresponding author on reasonable request.

## Electronic supplementary material

Supporting Information
