# Supplementary material for: Label-free multiphoton microscopy reveals relevant tissue changes induced by alginate hydrogel implantation in rat spinal cord injury
Source: Sci Rep. 2018 Jul 18;8:10841. doi: 10.1038/s41598-018-29140-z (PMC6052076; doi:10.1038/s41598-018-29140-z)
Supplement: Supplementary file 1 — Supporting Information [file 41598_2018_29140_MOESM1_ESM.pdf]

SUPPORTING INFORMATION

Label-free multiphoton microscopy reveals relevant tissue changes induced by alginate hydrogel implantation in rat spinal cord injury

Roberta Galli[1,†], Kerim H. Sitoci-Ficici[2,†], Ortrud Uckermann[2], Robert Later[2], Magda Marečková[2,4], Maria Koch[2,4], Elke Leipnitz[2], Gabriele Schackert[2], Edmund Koch[1,4], Michael Gelinsky[3,4], Gerald Steiner[1,*], Matthias Kirsch[2,4,*]

[1] Clinical Sensoring and Monitoring - Anesthesiology and Intensive Care Medicine, Faculty of Medicine, TU Dresden, Fetscher str. 74, 01307 Dresden, Germany

[2] Neurosurgery, University Hospital Carl Gustav Carus, Faculty of Medicine, TU Dresden, Fetscher str. 74, 01307 Dresden, Germany

[3] Translational Bone, Joint and Soft Tissue Research, Faculty of Medicine, TU Dresden, Fetscher str. 74, 01307 Dresden, Germany

[4] CRTD / DFG-Center for Regenerative Therapies Dresden - Cluster of Excellence, Fetscher str. 105, 01307 Dresden, Germany

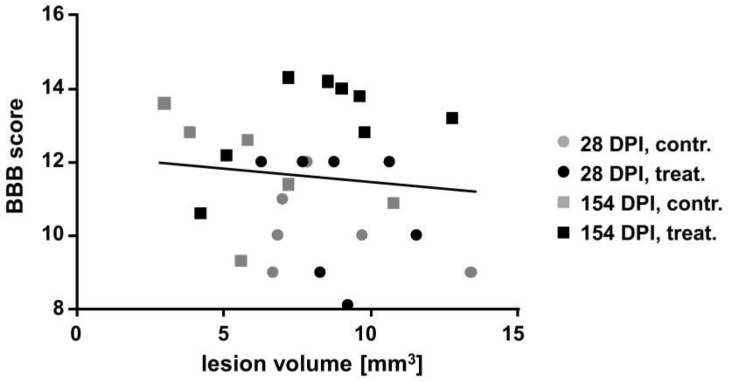

Supporting Figure S1. Scatter plot of ipsilateral hindlimb functions vs. lesion volume. BBB scores at 154 DPI are the average of scores obtained between 70 and 154 DPI. The slope of the linear fit is not significantly different from zero ($r^2 = 0.011$, $p = 0.61$).

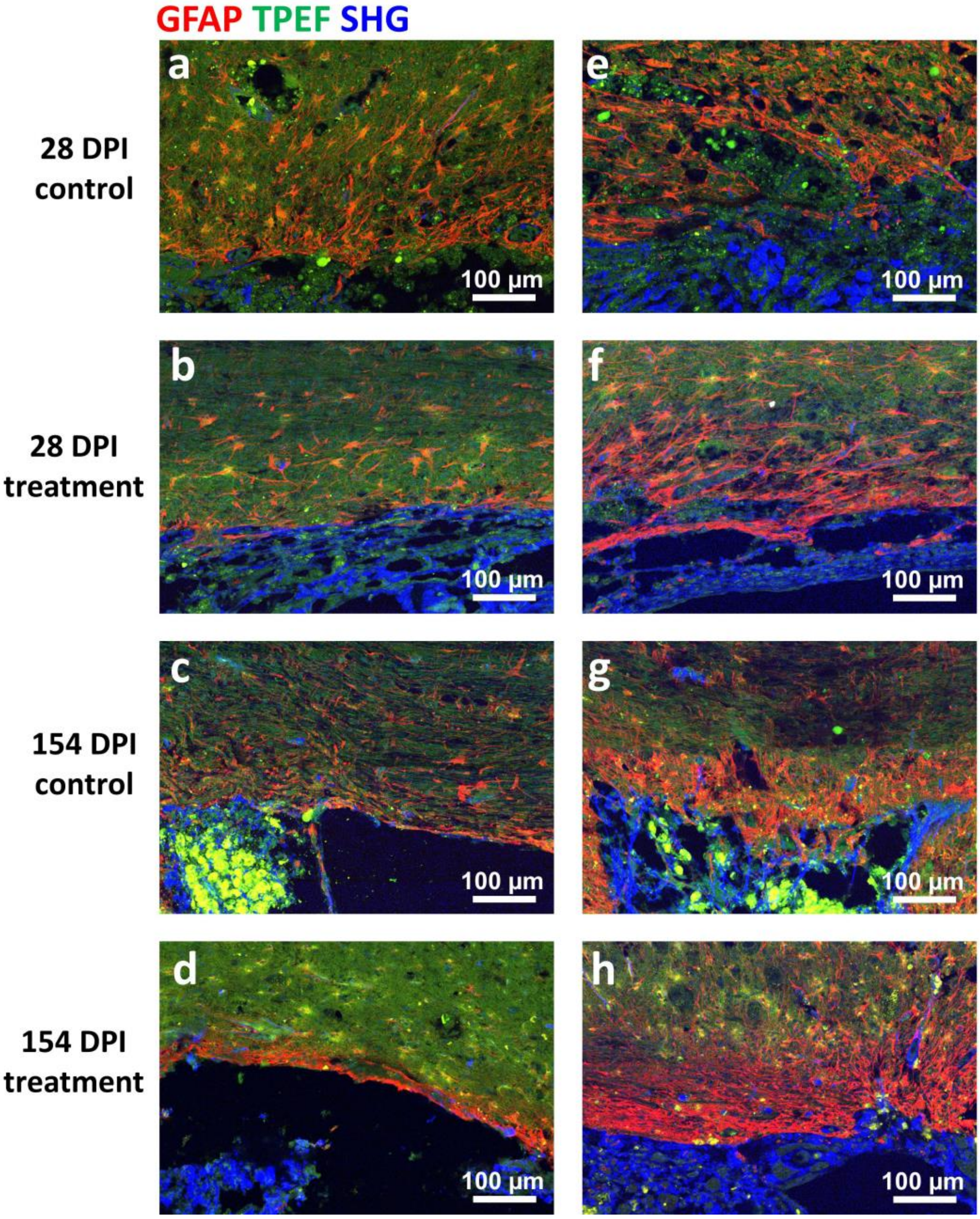

Supporting Figure S2. Glial scar visualized by GFAP immunohistochemistry. Multiphoton images of longitudinal sections of treated and control animals 28 and 154 DPI (red: GFAP, green: TPEF, blue: SHG). Images on the left (a-d) show regions with a thinner glial scar, and images on the right (e-h) show regions with a thicker scar. At 154 DPI, a very thin glial scar is typically observed in combination with a very thin fibrotic scar, and vice versa (compare c with g, and d with h).

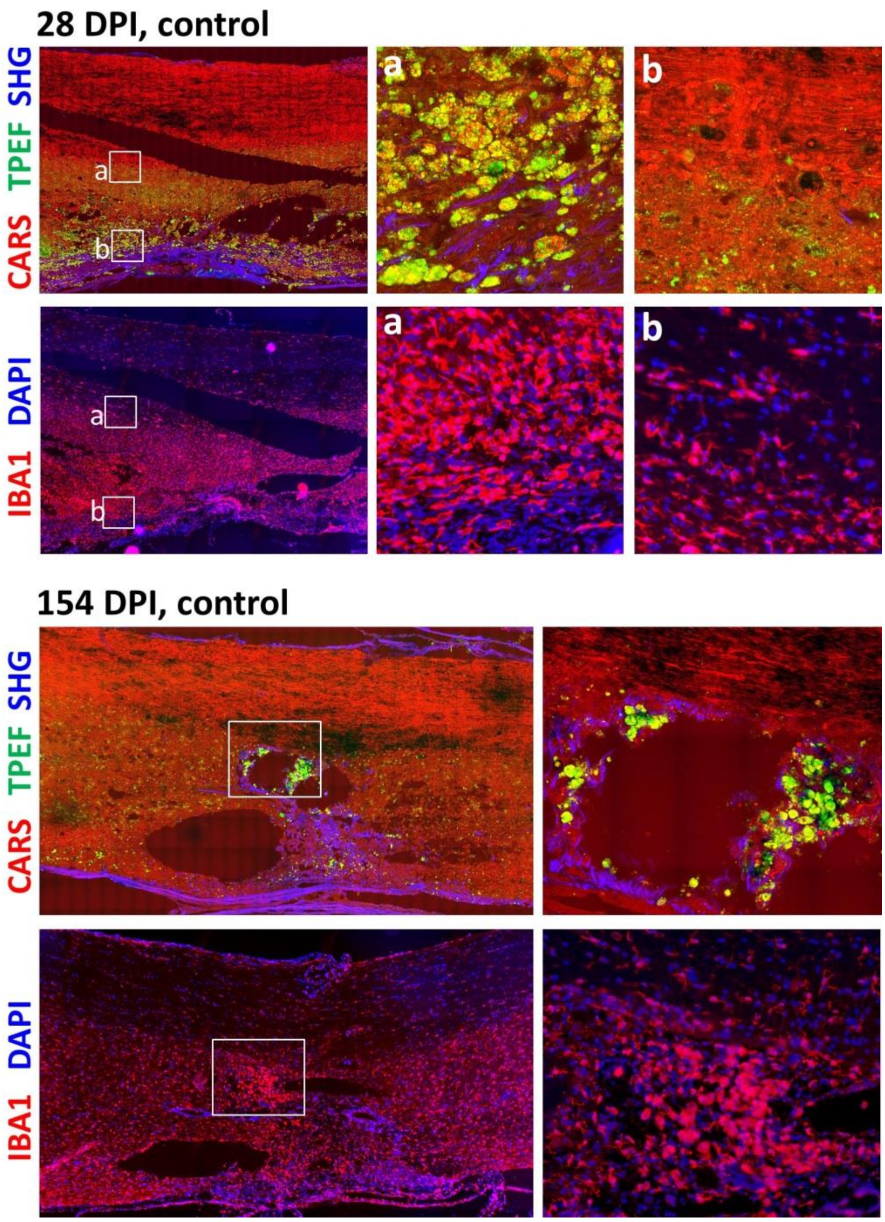

Supporting Figure S3. Comparison of multiphoton microscopy and IBA-1 microglial marker on two SCI control samples at 28 and 154 DPI. Regions with strong TPEF-positive cells with the morphology of foam cells correspond to regions where stained cells possess the amoeboid phenotype of activated microglia/macrophages. Regions that are mostly or totally devoid of TPEF positive cells correspond to regions where IBA1-stained cells display the ramified phenotype of resting microglia.

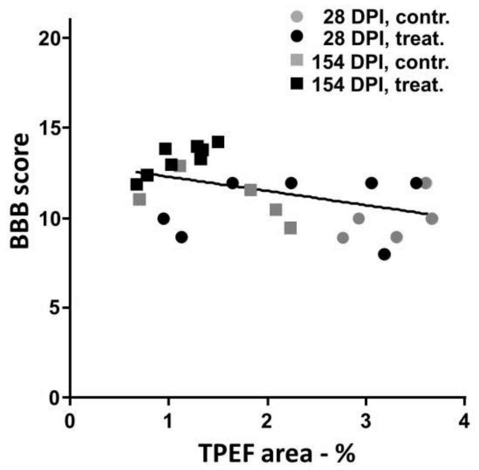

Supporting Figure S4. Scatter plot of ipsilateral hindlimb functions vs. TPEF area. BBB scores at 154 DPI are the average of scores obtained between 70 and 154 DPI. The degree of correlation is weak ($r^2 = 0.21$), although the slope of the linear fit is significantly different from zero ($p = 0.020$).

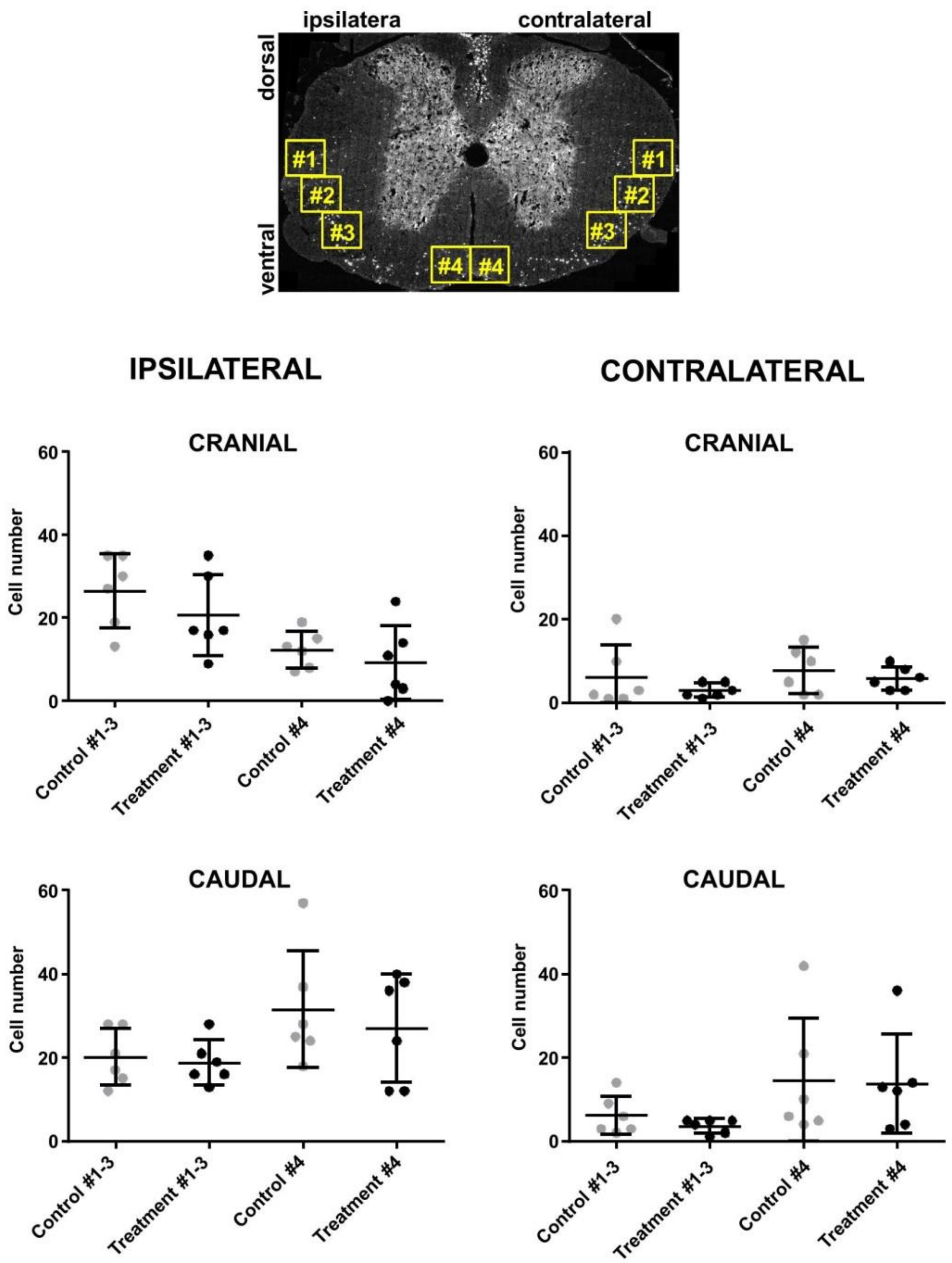

Supporting Figure S5. Distribution of inflammatory cells. Fluorescent cells were counted in four areas (#1 to #4) 210 µm × 210 µm large on the TPEF images of cross-sections. The values measured in areas #1 to #3 did not evidence any significant difference and were thus averaged.
